# Linking Electronic Health Records and In-Depth Interviews to Inform Efforts to Integrate Social Determinants of Health Into Health Care Delivery: Protocol for a Qualitative Research Study

**DOI:** 10.2196/36201

**Published:** 2022-03-11

**Authors:** Annemarie Hirsch, T Elizabeth Durden, Jennifer Silva

**Affiliations:** 1 Department of Population Health Sciences Geisinger Danville, PA United States; 2 Department of Sociology and Anthropology Bucknell University Lewisburg, PA United States; 3 Paul H. O'Neill School of Public and Environmental Affairs Indiana University Bloomington, IN United States

**Keywords:** electronic health records, social determinants of health, poverty, rural, qualitative, health system

## Abstract

**Background:**

Health systems are attempting to capture social determinants of health (SDoH) in electronic health records (EHR) and use these data to adjust care plans. To date, however, methods for identifying social needs, which are the SDoH prioritized by patients, have been underexplored, and there is little guidance as to how clinicians should act on SDoH data when caring for patients. Moreover, the unintended consequences of collecting and responding to SDoH are poorly understood.

**Objective:**

The objective of this study is to use two data sources, EHR data and patient interviews, to describe divergences between the EHR and patient experiences that could help identify gaps in the documentation of SDoH in the EHR; highlight potential missed opportunities for addressing social needs, and identify unintended consequences of efforts to integrate SDoH into clinical care.

**Methods:**

We are conducting a qualitative study that merges discrete and free-text data from EHRs with in-depth interviews with women residing in rural, socioeconomically deprived communities in the Mid-Atlantic region of the United States. Participants had to confirm that they had at least one visit with the large health system that serves the region. Interviews with the women included questions regarding health, interaction with the health system, and social needs. Next, with consent, we extracted discrete data (eg, diagnoses and medication orders) for each participant and free-text clinician notes from this health system’s EHRs between 1996 and the year of the interview. We used a standardized protocol to create an EHR narrative, a free-text summary of the EHR data. We used NVivo to identify themes in the interviews and the EHR narratives.

**Results:**

To date, we have interviewed 88 women, including 51 White women, 19 Black women, 14 Latina women, 2 mixed Black and Latina women, and 2 Asian Pacific women. We have completed the EHR narratives on 66 women. The women range in age from 18 to 90 years. We found corresponding EHR data on all but 4 of the interview participants. Participants had contact with a wide range of clinical departments (eg, psychiatry, neurology, and infectious disease) and received care in various clinical settings (eg, primary care clinics, emergency departments, and inpatient hospitalizations). A preliminary review of the EHR narratives revealed that the clinician notes were a source of data on a range of SDoH but did not always reflect the social needs that participants described in the interviews.

**Conclusions:**

This study will provide unique insight into the demands and consequences of integrating SDoH into clinical care. This work comes at a pivotal point in time, as health systems, payors, and policymakers accelerate attempts to deliver care within the context of social needs.

**International Registered Report Identifier (IRRID):**

DERR1-10.2196/36201

## Introduction

Health systems are under increasing pressure to assess and act on social determinants of health (SDoH), with widespread support from professional organizations including the American Academy of Pediatrics, the American Academy of Family Physicians, and the American Medical Association. In 2014, the Institute of Medicine (currently the National Academy of Medicine) recommended the documentation of core measures of social and behavioral domains in electronic health records (EHRs), including education, financial resource strain, and stress [1]. More recently, the National Academies of Sciences, Engineering, and Medicine (NASEM) presented guidance for how clinicians and health systems should use SDoH data when managing patients [2]. These calls to action have prompted health systems to accelerate efforts to integrate SDoH into care delivery. It is imperative that these efforts are informed by the voice of key stakeholders, including clinicians documenting in the EHR and the patients receiving care.

Data on SDoH are entered into EHR systems through multiple pathways, but data are not routinely or systematically collected [3]. Medical vocabularies, including International Classification of Disease (ICD) codes and Logical Observation Identifiers, Names and Codes capture SDoH (eg, ICD-10 code Z55.0: problems related to education and literacy), but the codes are not comprehensive, and they are underused [4,5]. Medicare reported that the ICD codes used to capture SDoH were used for only 1.6% of Medicare beneficiaries in 2019 [6]. EHR vendors are working with health systems to develop patient-reported tools to improve the capture of SDoH in structured fields, but there is little standardization regarding what data to collect [7]. Clinician notes, while more challenging to access and use than discrete EHR data, have recently emerged as a potentially important source of SDoH data [7,8], but this data source remains relatively underexplored.

Potentially more challenging than capturing SDoH is the ability to identify the social needs and chronic stresses that patients prioritize. As described by Alderwick and Gottlieb [9], while screening tools can reveal multiple SDoH, such as food and housing instability, a patient may perceive that her most pressing social need is to escape from a violent partner. Thus, it is not sufficient for health systems to only collect SDoH data; they must also adequately capture the patient’s perspective on these data [10].

In addition to identifying social needs, the NASEM report called for health systems to respond to social needs through adjustment (altering clinical care to accommodate identified social barriers), assistance (providing support in connecting patients with relevant social care resources), alignment (activities to work with existing social care assets in the community), and advocacy [2]. Health systems have already started implementing these activities, but research regarding the impact of these activities on health outcomes is limited. Importantly, the unintended consequences of these activities, including the potential for perpetuating bias and further marginalizing vulnerable populations, are poorly understood [2,11,12]. EHR data, particularly clinician notes, maybe a rich source of secondary data that provides some important insight into how clinicians are currently responding to social needs and the consequences of their responses.

This paper describes the protocol for a qualitative research study that merges discrete and free-text data from EHRs with in-depth interviews from among women living in rural and socioeconomically deprived communities in the Mid-Atlantic region of the United States. The objective of this study is to describe discrepancies between the medical record and patient narratives that could help identify gaps in documenting SDoH in the EHR, highlight potential missed opportunities for addressing social needs, and observe unintended negative consequences of documenting or responding to SDoH. This study leverages multiple perspectives to provide unique insight into the demands and consequences of integrating SDoH into clinical care.

## Methods

### Overview

This study is being conducted by an interdisciplinary team across three institutions, a health system (here forward referred to as Central Health), Bucknell University, and Indiana University. It is an ongoing qualitative study that combines EHR data from the health system with in-depth interviews of patients recruited by the university research teams. This study was approved by the institutional review boards (IRB) of all participating institutions (Bucknell University IRB approval number: 1920-123; Geisinger IRB approval number: 2017-0440).

### Study Participants and Recruitment

To be eligible for this study, individuals had to have received care, per self-report, from Central Health and therefore have a Central Health EHR. Participants also had to be self-identified women over the age of 18 years with less than a four-year college degree. We used stratified sampling to recruit both White women and women of color, including women who self-identified as Black, Latina, and Asian Pacific. We recruited women residing in counties in the Mid-Atlantic region of the United States that are ranked among the lowest in health indicators, including poor physical and mental health days, as well as teen births, preventable hospital stays, and incidences of violent crimes and injury deaths [13]. We first recruited respondents through convenience sampling, visiting venues such as laundromats, bars and gaming rooms, public libraries, grocery stores, bus stops, dollar stores, public community events, churches, and job training workshops. We then employed snowball sampling, asking for each participant to recommend one to two people who might also participate in the study.

### In-Depth Interviews

We conducted one-on-one interviews in-person until we needed to shift to remote interviewing, via video conferencing, due to the COVID-19 pandemic. The interviews probed women about their past and current medical issues; their interaction with Central Health; their mental health; their understanding of required treatment and barriers to compliance; their experiences of stress, violence, discrimination, and economic deprivation; their families, children, and social support systems; and their sense of self-efficacy, trust, and the future. The interview allowed for participants to share their perspectives in their own words, rather than through a standard survey format. The authors conducted about two-thirds of the interviews, with trained student research assistants completing the remaining ones. We compensated interviewees for their time with $50 in cash at the completion of the interview. Interviews were recorded with the permission of the participant and fully transcribed by a professional firm.

### Electronic Health Record Data

Central Health is a single, large, integrated health system serving a mix of rural and urban communities, including communities designated as Medically Underserved Areas [14]. The health system facilities include multiple inpatient hospitals and more than 125 primary and specialty clinic sites. The system uses Epic EHR software modules, including ambulatory, inpatient, surgery, emergency department, e-prescribing, computerized physician order entry, registration, and scheduling.

We extracted a set of discrete and free-text clinician notes on all consenting interview participants, matching participants to their record based on first name, last name, and date of birth (Textbox 1). We included participant data for any contact with the health system between 1996 and the year of the completed interview (between 2017-2021). Using these data, the health system research team created an EHR narrative, a free-text summary of the health of the participants and their interactions with the health system. We developed these narratives using a three-phase approach implemented by trained research assistants. First, the research assistant wrote a summary of the participant’s health and contact with the health system in consecutive order, going from the first health system contact to the last, using only information from the discrete data fields. Next, the team members enriched this summary of discrete data with direct quotes from clinician notes. Research assistants included notes if the text addressed an SDoH (eg, occupation, education, marital status, history of trauma, transportation challenges, etc) or if the notes clarified discrete data (eg, a patient who did not fill prescriptions because of lack of insurance coverage). Notes were enclosed in quotation marks or highlighted to distinguish them from information obtained from discrete EHR data. The team was trained to be over-inclusive of potential SDoH, including any reference to life circumstances beyond health conditions and health care.

Finally, the research assistant revised the summary so that, rather than being purely consecutive, chronic conditions (eg, chronic pain and mental health) that were managed over time were described in their own separate sections, enabling readers to obtain the breadth, depth, and longevity of these chronic issues. In a pilot study of this methodology, the principal investigator and each research assistant completed narratives for the same five patients and refined training and instructions to ensure a consistent approach to the narratives. The principal investigator at the health system provided a second review of all final narratives to ensure completeness and to confirm that no personal identifiers were included.

**Electronic health record data extracted for all interview participants.**

**Sociodemographic factors:** date of birth, race, ethnicity, health insurance

**Health behaviors:** smoking history, alcohol history, illicit drug history, sexual history

**Diagnoses:** diagnoses associated with clinical encounters, dates of diagnoses

**Medication:** medication orders (name, pharmacy class, dosage, indications), dates of medication orders

**Utilization:** dates of inpatient, outpatient, emergency department encounters, and telemedicine

**Free-text notes:** telephone encounters, health system letters, encounter notes, after-visit summary

### Analysis

Central Health and the universities developed a data-sharing protocol to facilitate the merging of the interview and EHR data with minimal risk to participant confidentiality. Under the authors’ guidance, two trained members of the research team coded each interview and EHR narrative using the qualitative software NVivo. We employed an approach called “flexible coding,” which consisted of reading and rereading the interview transcripts line by line and slowly building connections across the different sources of data [15]. We entered the coding process with a list of codes, or “nodes,” that we anticipated based on our review of the literature, but our list quickly expanded to include nodes that the participants, either directly or indirectly, emphasized as salient features and events in their social contexts, daily routines, and health care experiences, leading to approximately 200 nodes. We then used NVivo to systematically analyze relationships between nodes as well as frequencies of occurrences in the data. During this process, the second and third authors, with a team of trained students, created two to three-page narrative summaries, with direct quotes, of each participant’s life history from their interview transcript, providing quick “snapshots” of cases for our reference.

At the same time, we did a close reading and mapping of observations in the EHR, notes, and interviews across three major categories: SDoH; stories of “health” and health care utilization/experiences; and interactions between the patient and the clinician. We first documented the similarities and differences between the EHR and patient narrative within each participant’s case, and then compared across cases to refine our concepts, specifying the conditions and contexts that seemed to explain differences across cases, and repeating this process until we had identified a working set of social processes [16].

## Results

To date, we have interviewed 88 women, including 51 White, 19 Black, 14 Latina, 2 mixed Black and Latina, and 2 Asian Pacific Islanders. We have extracted the EHR data on all participants with available EHR data and completed the EHR narratives on 66 women. The women ranged in age from 18 to 90 years. We found corresponding EHR data on all but 4 of the interview participants. Participants had contact with a wide range of clinical departments, including family practice, obstetrics and gynecology, specialty care (eg, psychiatry, otolaryngology, infection disease, neurology, endocrinology, cardiology, nutrition services, and ophthalmology), surgery (eg, vascular, general), and emergency medicine. Clinical visits occurred at the main campus of the health system, as well as community practice sites and smaller affiliated hospitals. Free-text notes were documented by different clinician types, including physicians, nurses, and social workers.

A preliminary review of the paired interviews and EHR narratives revealed that the clinician notes were a source of data on a range of SDoH (Textbox 2). Clinicians documented topics such as education and occupational history, transportation challenges, financial burdens, social isolation, issues with insurance, and childhood trauma. The notes also include data on how clinicians responded to social risk factors, including transportation issues and financial strain (Textbox 2). However, the notes did not always comprehensively capture the social needs of patients, as reflected in the misalignment of the social needs documented in the notes and the social needs described by patients during the interviews (Multimedia Appendix 1). Some interviewees reported that they withheld information from their clinicians out of fear or shame. Other interviewees attested to sharing information with their clinicians but felt that their clinicians did not take them seriously, resulting in perceptions of misdiagnosis and breakdowns in communications.

**Selected quotes from electronic health record notes regarding social determinants of health.**

**Financial strain:**

- “Patient does not have inhalers at home. Does not particularly like and also cost is an issue.”
- “Does not feel it (antidepressant) would help her as a lot of her mood changes are due to financial situation.”
- “Has financial burdens due to having to take care of the grandchildren and feed them for years.”

**Insurance:**

- “…she stopped getting them (mammograms) anymore because her insurance stopped paying.”
- “Due to transportation difficulties and lack of insurance, she only attended two prenatal appointments.”
- “During one of the visits this year, *the patient* noted that she had been doing well on Lipitor years ago for cholesterol but had to go off of it due to a change in her insurance. She requested to go back to Lipitor now that she had better health insurance.”

**Family trauma:**

- “Sexually abused by uncle as child and ‘gang raped’ at age 14.”
- “C/o ongoing depression… Worsening over the past year. Feels it started seven years ago after her child of 1 month and 20 days old died from SIDS. Also grew up in foster care and suffered physical and emotional abuse.”
- “Conflict in the home between mom and dad has increased significantly, with *patient* witnessing most of it. On occasion, she has gotten between mom and dad. Mom concerned about impact on *patient*.”
- “During this evaluation, it was revealed that *the patient’s* first husband had verbally and physically abused her prior to their divorce in 2003. As of 2012, *the patient* is married and lives with her husband and 2 children. Her husband also suffers from chronic pain, and her husband’s brother, who suffers from alcohol abuse, lives with them.”

**Clinician response to social risk factors:**

- “Pt… planning to apply for food stamps. Enrolled in WIC. Provided contact info for local pregnancy support programs and childcare subsidy program. Pt declined referral to Nurse/Family Partnership program.”
- “I talked with *patient* about transferring her to a more long-term, supportive therapist in her community. It has been difficult for her to attend regular appointments d/t to the distance.”
- “Has necessary baby supplies, except care seat, discuss rental from L+D unit.”

## Discussion

### Principal Findings

This is an innovative study that brings together two data sources, EHR data and patient voices, to help inform the integration of SDoH into health care delivery. Examining how these two data sources converge and diverge, we will identify successes as well as missed opportunities for capturing and addressing social needs and gain insight into the unintended consequences of these efforts. The goal of this study is to provide guidance to health systems as to how to collect and respond to SDoH.

The participants in the study are women with less than a college education, living in rural communities with high levels of community socioeconomic deprivation. We designed the study so that close to half of the sample was Black, Latina, or both Black and Latina. We selected a study population with these characteristics as this population suffers from a lack of health equity and is thus most likely to benefit from better integration of SDoH into medical care. Individuals living in rural communities, for example, are at an increased risk of death from five leading causes (heart disease, cancer, unintentional injury, chronic lower respiratory disease, and stroke) compared to their urban counterparts [17]. Among a large list of disparities for those marginalized racially and economically, life expectancy is shorter among Black individuals compared to White as well as residents of low-income communities compared to high-income communities [18]; infant mortality rates are higher in Black mothers compared to White mothers and among mothers with a high school education compared to those with a college degree; pregnancy-related mortality ratios are higher among Black women compared to White women [19]. This study provides a voice for individuals most likely to experience social barriers to good health. By enrolling White, Black, Latina, and Asia Pacific women, we are also primed to evaluate differences in health system interactions by race and ethnicity.

This study has multiple strengths. First, the study integrates two perspectives through the analysis of EHR data and patient experiences. Second, the study uses more than 25 years of EHR data, enabling the study team to examine social risk factors across the lifespan in a subset of women. Finally, we have developed a novel approach to analyzing EHR data through the development of an EHR narrative based on discrete and free-text data for qualitative analysis. The use of EHR data from a single health system may be a limitation of this study. However, the health system employs more than 1000 physicians and more than 900 advanced practitioners; therefore, the EHR data used in this study is from a range of clinicians in a variety of clinical settings. Our inability to describe the sociodemographic characteristics of the clinicians is a further limitation, given the growing body of literature on the importance of physicians’ characteristics to patients’ health experiences [20,21].

### Conclusions

As health systems accelerate attempts to integrate SDoH into the EHR and health care decisions, it is essential that these efforts are informed by the experiences of the patients receiving care, particularly the most vulnerable patients. This study will provide unique insight into the needs and consequences of integrating SDoH into clinical care. This work comes at a pivotal point in time, as health systems, payors, and policymakers accelerate attempts to deliver care within the context of social needs.

## Appendix

Multimedia Appendix 1 Sample electronic health record and interview narratives.

Multimedia Appendix 2 Peer-review report by the Russell Sage Foundation.

## Abbreviations

EHR: electronic health record
ICD: International Classification of Disease
IRB: institutional review board
NASEM: National Academies of Sciences, Engineering, and Medicine
SDoH: social determinants of health
