# Supplementary material for: Linking Electronic Health Records and In-Depth Interviews to Inform Efforts to Integrate Social Determinants of Health Into Health Care Delivery: Protocol for a Qualitative Research Study
Source: JMIR Res Protoc. 2022 Mar 11;11(3):e36201. doi: 10.2196/36201 (PMC8956985; doi:10.2196/36201)
Supplement: Multimedia Appendix 1 [file resprot_v11i3e36201_app1.docx]

**Multimedia Appendix 1**

**Sample EHR Narrative (abridged) – Giselle (pseudonym)**

Giselle, according to her EHR, is a “white female” and former smoker who has been diagnosed repeatedly with “persistent depressive disorder and anxiety disorders, teeth disorders, and morbid obesity due to excessive calories”. She was prescribed the generic version of Zoloft for one year. The physician’s notes delve into the traumas she has suffered and her complicated relationships: “C/o ongoing depression x years. Worsening over the past year. Feels it started seven years ago after her child of 1 month and 20 days old died from SIDS. Also grew up in foster care and suffered physical and emotional abuse.” Subsequent notes on two visits record a “referral to outpatient psychology” and explain that “During the visit the clinician also noted that Giselle wanted to lose weight. Giselle reported dieting and exercise with no results. She reported obesity in the family and was interested in medication to lose weight.” Giselle was then supervised for a high risk pregnancy, where it was noted in her chart that she had “mental disorders, obesity, and coagulation defects.”

One year later, Giselle was hospitalized for an infection and underwent a psychiatric evaluation. The notes explain that she was “a little depressed as she has been cyber bullied recently due to her weight. The police are aware and are involved with the situation. She states she has removed herself from this situation.” The notes also document her “occasional suicidal thought, but has no plan and this was due to severe tooth and mouth pain which has gone untreated for approx 4 months,” but “the pain is becoming more bearable due to treatment.” The notes again document her childhood history of physical and emotional abuse in foster homes (learned during a screening for violence), as well as the short duration of taking Zoloft for depression (as she has not had the prescription refilled) and her lack of attendance at outpatient care for counseling:

“She has never had formal counselling for this trauma and admits to some depression due to same for which her OB/GYN gave her Zoloft but this was stopped during pregnancy. She did restart it for a short period once she delivered her sons but has not taken it for several months now as she has not had the prescription refilled. Denies out pt psychiatry care. She would like to start a formal program for out pt counseling to help with her traumatic upbringing and also to help her deal with the bullying issue that is ongoing at present.”

Her last note, when she is 29, comes from hernia surgery where it was noted that “Giselle worked at a paper factory where she lifts heavy materials throughout [the] shift. She also reported that she had 25 lb twin boys who she occasionally lifted. She also reported that she was going through a divorce. She also noted that she was considering bariatric surgery for her weight. The clinician discouraged this option and gave a referral to a nutrition-clinical dietitian.”

**Sample Interview Narrative - Giselle**

During her interview, Giselle shares personal information that, if integrated into the EHR, could potentially lead her clinicians to adjust their recommendations for treatment. Major themes include extreme financial strain, the need to choose between having enough money to survive or seeking medical treatment, and her conviction to put her children’s needs before her own. Giselle shared that she was abandoned by her boyfriend when she was pregnant with her first child. Giselle then got married to another man, had twin boys, and got divorced. Finances have always been a source of anxiety, especially because Giselle fears that her children might be taken away if she loses her job: “My kids come before me. If I can keep them out of foster care, I’m going to…I wish my parents felt the same way.” She has no savings and does not own her home. She recalls that, “before I had gotten on state insurance, all my co-pays would always go unpaid. I am always fighting to pay bills now, let alone have to find the $150 for an ER visit.” Giselle also notes the lack of support from the people around her: “I have family and they live all around but they wouldn’t do anything for me if I asked them to.”

In the past, she has lost jobs because of her health – she had to quit a stocking job when she was put on bed rest during her high risk pregnancy and she “got fired, pretty much” from a job at a technology plant after her recent hernia surgery because “you need to be on light duty for two weeks after.” She finally has a steady job making $16 an hour but she still receives “WIC and food stamps to make sure my kids are fed, barely making ends meet with my paycheck.” As a child, she recalls, she was bullied for using food stamps, which was a source of shame.

When one of Giselle’s children was very young, this child was often ill, and had to have surgery on his skull. Giselle describes, “he was in the NICU from the time he was born” and “had this part of his forehead cut out and reconstructed and put back in. He has a scar that goes from ear to ear.” Giselle states that, without Medicaid, she “wouldn’t have been able to afford his skull surgery which was $115,000. I had to pay, like 600.” Describing this child’s current medical challenges, Giselle adds: “my son’s medicine is $200 a month. Even with my ex-husband’s insurance we still have to pay $30 out of pocket for his meds.”

Giselle describes limiting her own doctor’s visits and prescriptions to save money for her children’s needs. She explains, “My doctor wants me to see a nutritionist and I do want to lose weight but it’s on the back burner. I didn’t go yet. It was around the time that she [her daughter] got sick and I want to make sure she’s okay before I take care of myself. That’s how I’ve always been with all three of my kids.” She also notes that she leaves the house at 4:30 in the morning to drop off her children at daycare and get to work by 5:30am, often working ten hour days, leaving little time for herself. While weight loss, mental health counseling, and prescriptions for her depression are recurring issues in the EHR, Giselle says simply, “I try to do at least 15,000 steps a day.” Giselle also mentions that, while she is happy with her children’s health care, the doctors she has seen tend to rush her, giving off the vibe “okay, I’ll listen but can you speed up? I want to get home.”

| **Table A. Giselle’s interaction with health system and social determinants of health from two data sources: electronic health record and patient narrative** | | |
| --- | --- | --- |
|  | **Electronic health record narrative** | **Patient narrative** |
| Clinical problems and treatments | Former smoker, previous alcohol abuse | Former smoker, now vapes as stress reliever  Drinks occasionally |
|  | Teeth disorder, Ludwig Angina, mouth pain. Stayed in hospital for 3 nights. | Teeth disorder, Ludwig Angina, mouth pain. In intensive care unit for 5 days. |
|  | Morbid obesity | Wishes she could lose weight |
|  | Abdominal pain, ventral hernia – removed | Gallbladder surgery |
|  | Thrombocytopenia (low blood platelet count) | C-section, postpartum depression |
|  | Depression | Depression, anxiety, was on antidepressants |
| Interaction with clinicians | At the age of 26, Giselle returned to family practice twice. | She is incredibly thankful and impressed with the care her children have received. She says, “that doctor seems like he really cares about the children.” She mentions that doctors she has seen tend to rush her, giving off the vibe “okay, I’ll listen but can you speed up? I want to get home.” |
|  | “OB/GYN gave her Zoloft but this was stopped during pregnancy. She did restart it for a short period once she delivered the twins but has not taken it for several months now as she has not had the prescription refilled.” | Giselle also wishes that doctors would spend more time in the room with their patients. She feels like doctors are disconnected from patients and are oftentimes in a “quick rush” -- they come in, send stuff away for tests, make you wait between thirty minutes and an hour, come back, tell you what’s wrong, and leave. |
|  | She also noted that she was considering bariatric surgery for her weight. The clinician discouraged this option and gave a referral to a nutrition-clinical dietitian. | “He wants me to see a nutritionist, and I do want to lose weight, but it’s on the back burner.” |
| Social determinants of health | “Feels it started seven years ago after her child of 1 month and 20 days old died from SIDS. Also grew up in foster care and suffered physical and emotional abuse. Denies sexual abuse. Talks to her sister a lot. Sister has recommended she get treatment. Sister is on medication for depression. Pt c/o stress with family and friends. Talking behind her back. Calling names. Feels bad about herself.” | Receives WIC and food stamps. She sees food stamps as somewhat stigmatized - used to be bullied in school because her family received them. |
|  | “Has personally or witnessed someone else being physically abused including, but not limited to, being punched, slapped, kicked, strangled, restrained, burned, threatened with object or weapon, etc.” | “I’m barely making ends meet with my paycheck and I would like my kids to be fed.” |
|  | During this visit it was noted that Giselle worked at a factory where she lifts heavy materials through our shift. | “Before I got state insurance, all of my copays would always go unpaid.” |
|  |  | “I have family and they live all around but they wouldn’t do anything for me if I asked them to.” |
|  |  | She has to lift heavy materials throughout her shift. |
