# Supplementary material for: Linking Electronic Health Records and In-Depth Interviews to Inform Efforts to Integrate Social Determinants of Health Into Health Care Delivery: Protocol for a Qualitative Research Study
Source: JMIR Res Protoc. 2022 Mar 11;11(3):e36201. doi: 10.2196/36201 (PMC8956985; doi:10.2196/36201)
Supplement: Multimedia Appendix 2 [file resprot_v11i3e36201_app2.pdf]

INSTRUCTIONS: As part of your application, please respond to the comments on your application, if applicable. Go to the "Review and Responses" box at the top of your application, download the Word document containing the reviews for this project, respond to the reviews in the same document by providing a point-by-point response to

JENNIFER SILVA

Linking Electronic Health Records and In-depth Interviews to Uncover Barriers to Social Mobility and Health in a Declining Coal Mining Community

Fluxx Request ID: R-1905-15244

Reviewer #1:

This is an important project meant to use multiple levels of data—in depth interviews and electronic medical records—to explore how locale, race and poverty impact health and how health concerns inhibit social mobility. This is primarily a qualitative project that will expand the Foundation's work centered on rural populations. The researchers provided a detailed and convincing review of the current literature on white despair in rural America and the increasing radicalization of these areas. The proposed project will focus on the coal region of Pennsylvania where these factors are on display and can be leveraged along with the electronic medical records to provide new insights about health and social mobility. The addition of 40 women of color to those being interviewed will expand their findings from the preliminary research, allowing them to pursue a more intersectional analysis with a focus on race instead of only whiteness. Professor Silva's past publication record, in particular her book "Coming Up Short" suggests that she has a nose for important questions and populations and the skill set to execute a novel and insightful research project. I think the proposed LOI outlines significant work and I would support requesting a full proposal.

Investigator Response

We thank Reviewer #1 for their careful reading of our proposal and the support of our research.

Reviewer #2:

This project is very promising, and important. In the event a full proposal is invited, I would recommend a few things. First, a strong proposal would have a strong justification for the qualitative sample size and composition. Whatever the size is, we would want to know why it was chosen. The inclusion of a diverse group of participants, for example, as described in the LOI, would be part of a good justification. Second, I recommend some focus with respect to the questions. The themes in this project---patient-doctor relations, trust in the system, barriers to health, socio-economic differences, cultural obstacles, etc.---are all interesting and important. However, themes are not questions. A strong proposal would be clear on what precise questions the project will answer.

Investigator Response

We thank Reviewer #2 for their careful review of our proposal. In regards to the sample size, 40 white women have already been interviewed. With support from Russell Sage, we will add 40 black and Latina women to this sample. We chose to aim for a total sample size of 80, which is small enough to do justice to the depth of meaning and the vividness of the data while large enough to establish patterns, look for cases that challenge our theoretical presumptions, and draw robust comparisons between the different groups.

Thank you for pushing us to more carefully consider the concrete research questions. Our principle research questions are as follows:

1. What are the mechanisms, as identified in rural women's narratives of their experiences and behaviors, through which economic disadvantage and racial identity impact their physical and emotional well being?
    a. How do these mechanisms vary by race?
2. What kinds of discrepancies exist between the formal medical record and patient narratives in terms of sources of poor health, diagnoses, and necessary treatment plans?
    a. How do these mechanisms vary by race?
3. How do experiences within the healthcare system itself shape women's everyday approaches to their own health and well being, as narrated by women themselves?
    a. How do these mechanisms vary by race?
4. Can we identify possible patterns in diagnoses, prescribing of medications, and creation of treatment plans in the electronic health records that might either promote or hinder participants' ability to achieve economic and social well being?
    a. Are there differences by race?
